# The clinical value of nomogram combined with machine learning models in predicting the progression of hypertensive disorders in pregnancy to severe preeclampsia

**DOI:** 10.3389/fgwh.2026.1672534

**Published:** 2026-02-12

**Authors:** Xiaoming Wang, Xingcheng Mao, Chao Xing

**Affiliations:** 1 Department of Clinical Laboratory, Wenzhou People’s Hospital, Wenzhou Women and Children’s Hospital, Zhejiang, China; 2 Department of Laboratory Medicine, the Second Affiliated Hospital and Yuying Children’s Hospital of Wenzhou Medical University, Zhejiang, China

**Keywords:** hypertensive disorders of pregnancy, machine learning, nomogram, predictive model, severe preeclampsia

## Abstract

**Background:**

Hypertensive disorders in pregnancy (HDP) include gestational hypertension, preeclampsia, and eclampsia. Not all cases of gestational hypertension or mild preeclampsia progress to severe conditions. However, once they develop into severe preeclampsia (SPE), the risks to both the mother and the fetus increase significantly. We aimed to establish a nomogram and train a machine learning (ML) model that could identify SPE, early in the course of HDP.

**Methods:**

In this retrospective study, 593 patients with HDP were enrolled in the training cohort. For predicting SPE early, six supervised ML models were employed, such as XGBoost, K-nearest neighbors (KNN), random forest (RF), LightGBM (LGBM), Support Vector Machines (SVM), and Decision Trees (DT), which were evaluated by accuracy (ACC) and the areas under the receiver operating characteristic curve (AUC). The nomogram was established, and the predictive ability was assessed by AUC, the calibration curve and clinical decision curves (DCA). They were validated by a validation cohort of 255 patients with HDP.

**Results:**

The nomogram model achieved an AUC of 0.934 in the training cohort, with a calibration curve Brier score of 0.083 and a clinical applicability probability threshold of 5%–95%. In the validation cohort, it showed an AUC of 0.882, a calibration curve Brier score of 0.115, and a clinical applicability probability threshold of 10%–95%. In the validation cohort, the AUC of XGBoost, KNN, RF, LGBM, SVM, DT, and multivariate logistic regression analysis models were 0.876, 0.822, 0.866, 0.866, 0.871, 0.784, and 0.847, the XGBoost model showed the highest AUC.

**Conclusions:**

This study demonstrates that a family history of hypertension, urine protein, umbilical artery S/D ratio, WBC, TBIL, UA, LDL, TG, CRP, and blood Ca are predictors of HDP progression to SPE. A nomogram model for predicting the progression of HDP to SPE was constructed using these predictors. The model exhibited good discrimination, calibration, and clinical utility in both the training and validation cohorts. Additionally, a ML model was developed, with the XGBoost model identified as the optimal one, which can be applied clinically in conjunction with the nomogram prediction model.

## Introduction

Hypertensive disorders of pregnancy (HDP) are a group of conditions characterized by the coexistence of hypertension and pregnancy, with a global incidence of approximately 7%–12%. These disorders are a major contributor to maternal mortality, ranking as the second leading cause of maternal death (1, 2). The American College of Obstetricians and Gynecologists (ACOG) defines HDP as systolic blood pressure ≥140 mmHg or diastolic blood pressure ≥90 mmHg measured at least twice, four hours apart, in pregnant women (3). Preeclampsia (PE), a subtype of HDP, is primarily characterized by proteinuria and hypertension. Due to its multifactorial and complex nature, its global incidence ranges from 2% to 8% (4). Severe preeclampsia (SPE) is diagnosed when PE is accompanied by systolic/diastolic blood pressure ≥160/110 mmHg, hemolysis, elevated liver enzymes, or low platelet count. The progression to SPE significantly increases the risk of fetal death, with a reported mortality rate of approximately 2% (5). Without timely detection and intervention, severe adverse outcomes such as maternal and fetal death may occur. Early prediction of the disease can help safeguard maternal and fetal health (6). Crucially, the pathogenesis of PE remains incompletely understood, involving multiple factors related to the fetus, placenta, and maternal system. The widely accepted two-stage theory proposes that the first stage involves inadequate remodeling of spiral arteries by trophoblasts, leading to reduced placental perfusion (7). Lunell et al. (8) reported a 50% reduction in uteroplacental blood flow in PE patients, with this decrease being more pronounced in SPE compared to mild PE. The second stage involves maternal endothelial dysfunction, where an imbalance between pro-angiogenic and anti-angiogenic factors contributes to the disease's manifestation (7). In animal models of PE, Alexander et al. (9) observed increased circulating levels of soluble vascular endothelial growth factor receptor-1 (sVEGFR-1) and elevated pro-inflammatory cytokines such as tumor necrosis factor-alpha (TNF-α) and interleukin-6 (IL-6). Therefore, the development of an accurate predictive model to identify patients at high risk of developing severe preeclampsia is of paramount clinical importance.

A nomogram is a graphical tool that enables personalized risk assessment and facilitates clinical decision-making (10). XueFei Liu et al. (11) constructed a nomogram to predict the progression of HDP to PE by integrating radiomic features, maternal age, and body mass index (BMI). The results demonstrated excellent predictive performance in both the training cohort [AUC of 0.89 (95% CI, 0.82–0.95)] and the test cohort [AUC of 0.85 (95% CI, 0.73–0.97)]. Shan et al. (12) identified five common predictive factors, BMI, urine protein, uric acid, age, and mean arterial pressure, through multivariate logistic regression analysis. The model showed strong discriminative ability in both the training cohort (AUC = 0.910) and the validation cohort (AUC = 0.890) for predicting HDP. In addition, machine learning methods have recently emerged as a research hotspot, assisting in early disease diagnosis by analyzing and learning complex patterns from medical data. Machine learning encompasses supervised learning algorithms such as support vector machines (SVM), random forests (RF), k-nearest neighbors (KNN), naive Bayes, and neural networks, as well as unsupervised learning algorithms like clustering and dimensionality reduction (13). S Schmidt et al. (14) developed two prediction models based on the GBDT and RF algorithms using placental growth factor, soluble FMS-like tyrosine kinase-1, and ultrasound indicators. The GBDT model was found to outperform the RF model in predictive efficacy. However, to the best of our knowledge, there remains a scarcity of established machine learning models specifically targeting SPE prediction.

Our study aims to construct and compare machine learning models and nomogram models for predicting the occurrence of SPE using laboratory indicators and ultrasonographic markers. The results may contribute to mitigating the progression of HDP, thereby improving maternal and neonatal outcomes.

## Materials and methods

### Study design and patients

**Figure 1:**
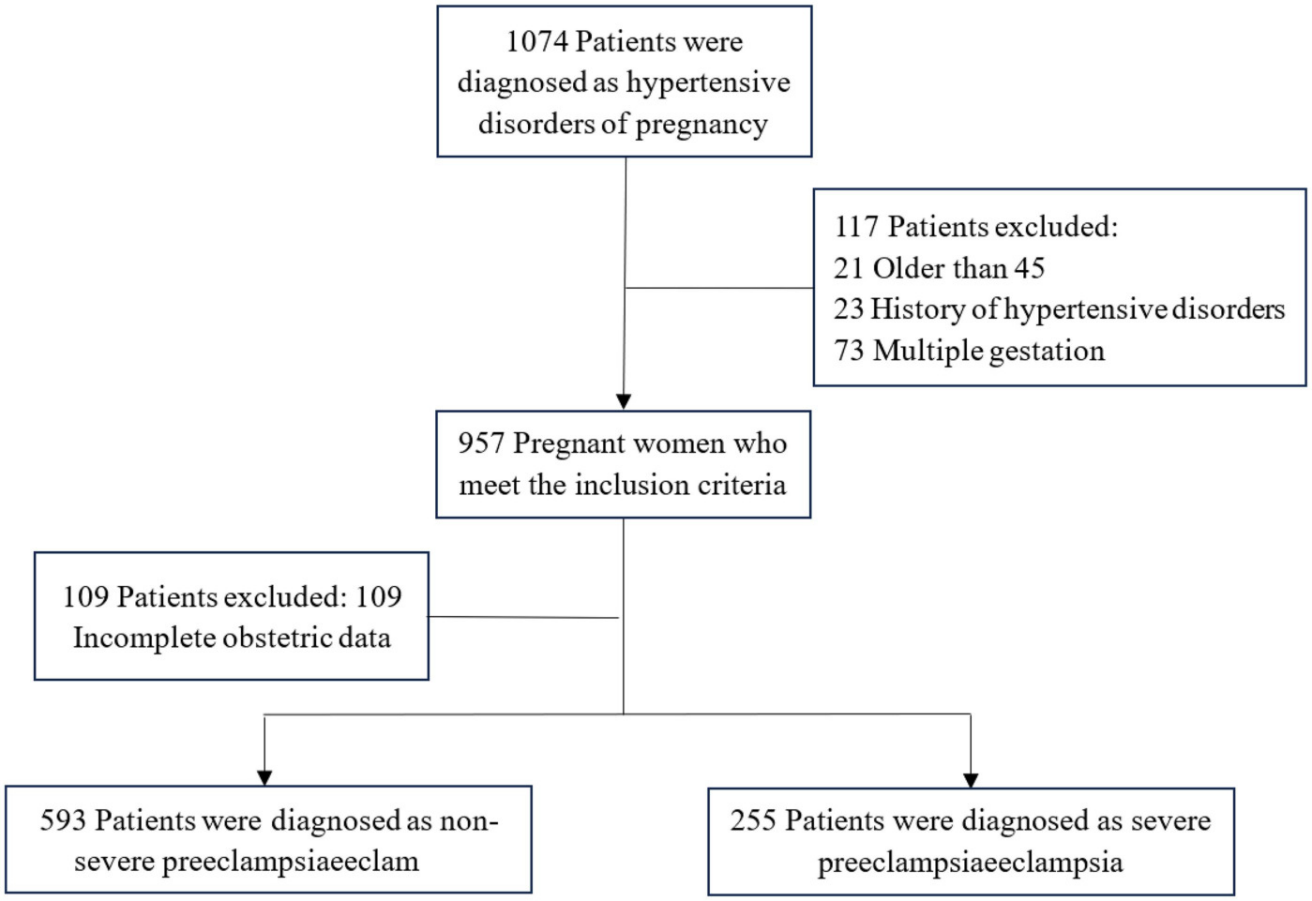
Flow diagram of patients selection.

This was a single-center, retrospective, observational cohort study. Clinical data of 1,074 HDP patients were collected and assessed from the Hospital Information System database in Wenzhou People's Hospital between January 2017 and January 2022. The hypertensive disorders in pregnancy examined in this study are classified into three categories, with the diagnostic criteria as follows, (1) Gestational Hypertension: hypertension first occurring after 20 weeks of pregnancy, with systolic/diastolic blood pressure ≥140/90 mmHg, returning to normal within 12 weeks postpartum, and no proteinuria; (2) Preeclampsia: gestational hypertension accompanied by either positive proteinuria, a urinary protein/creatinine ratio ≥0.3, or urinary protein ≥300 mg/24 h; or, in the absence of proteinuria, evidence of end-organ damage (such as thrombocytopenia, abnormal liver or kidney function, pulmonary edema, neurological symptoms, visual disturbances, etc.); (3) Severe Preeclampsia: preeclampsia with any of the following: blood pressure ≥160/110 mmHg, severe end-organ dysfunction, or involvement of the placental-fetal unit. Among the 1,074 patients, 226 patients were excluded based on the study exclusion criteria, which included (1) older than 45; (2) lack of data at admission; (3) history of hypertensive disorders; and (4) multiple gestation. Eventually, 848 eligible patients were enrolled in this study, including 593 patients with non-severe preeclampsia and 255 patients with severe preeclampsia (Figure 1). The study design was approved by the Research Ethics Board of the Wenzhou People's Hospital, and all analyses were performed following the Declaration of Helsinki.

### Data collection and definitions

Clinical data, including demographic characteristics, the clinical history, laboratory indicators, and ultrasound indicators. Demographic characteristics included weight, height [to calculate body mass index (BMI)], baseline systolic blood pressure and diastolic blood pressure [to calculate mean arterial pressure (MAP)], age. Clinical history included family history of hypertension, thyroid disorders during pregnancy, gestational diabetes mellitus, whether it is the first delivery, and history of adverse pregnancy. Laboratory indicators included urine protein (UP), white blood cell count (WBC), neutrocyte (Neut), lymphocyte (Lymph), red blood cell count (RBC), hemoglobin (HGB), hematocrit (HCT), mean corpuscular volume (MCV), mean corpuscular hemoglobin (MCH), mean corpuscular hemoglobin concentration (MCHC), platelet (PLT), platelet hematocrit (PCT), prothrombin time (PT), international normalized ratio (INR), automated partial thromboplastin time (APTT), fibrinogen (FIB), thrombin time (TT), d-dimer (DD), fibrinogen degradation product (FDP), total bilirubin (TBIL), total protein (TP), uric acid (UA), alkaline phosphatase (ALP), glutamate transpeptidase (GGT), low-density lipoprotein (LDL), triglycerides (TG), C-Reactive Protein (CRP), β_2_-microglobulin (β2-MG), aspartate aminotransferase (AST), creatinine (CR), alanine aminotransferase (ALT), homocysteine (HCY), and blood calcium (Ca). Ultrasound indicators included the umbilical artery blood flow S/D ratio.

### ML models and nomogram

We randomly divided the data into training and validation cohorts in a 7:3 ratio. The training cohort was used to build the model, while the validation cohort was used to evaluate it. Univariate analysis and LASSO regression analysis were applied to the training cohort to preliminarily screen for influencing factors of SPE. These factors were then incorporated into a multivariate logistic regression analysis to identify predictors of SPE, and a nomogram was constructed to complete the modeling. The model's performance was assessed using ROC, calibration curve, and clinical decision curve.

Additionally, the Boruta feature selection method was employed to identify important variables. We selected Decision Trees (DT), LightGBM, Support Vector Machines (SVM), XGBoost, Random Forest (RF), and K-Nearest Neighbors (KNN) as classifiers to predict severe preeclampsia, thereby establishing six machine learning models. The model's performance was assessed using accuracy (ACC) and ROC.

### Statistical analyses

Statistical analyses in this study were performed using the R version 4.2.1 and Python version 3.8. The “comparegroups” package was used for baseline description and comparative analysis. Normally distributed continuous variables were expressed as mean ± standard deviation (SD), nonnormally distributed continuous variables as median (interquartile range, IQR), and categorical variables as counts (%). Categorical variables were compared using the chi-square test, while continuous variables were analyzed using the independent samples *t*-test or Mann–Whitney *U*-test. The package automatically identified data types and applied the appropriate statistical method, results with *P*-value <0.05 were considered statistically significant.

Univariate logistic regression analysis and multivariate logistic regression analysis was conducted using the “glm” function, and LASSO logistic regression was carried out using the “glmnet” package. The nomogram and calibration charts were generated using the “rms” package. ROC curves were plotted using the “pROC” package, and the DCA curves were constructed using the “rmda” package. Boruta feature selection was conducted using the “Boruta” package. RF analysis was performed using the R package “randomForest”, KNN analysis was performed using the R package “kknn”, DT analysis was performed using the R package “rpart”, XGBoost analysis was performed using the R package “xgboost”, SVM analysis was performed using the R package “e1071”, LightGBM analysis was performed using the R package “lightgbm”, multivariate logistic regression analysis was performed using the R package “glm”. Comparison of ROC curves between different models was performed using the R package “ROCR”.

## Result

### Baseline characteristics

Overall, a total of 848 patients from the Hospital Information System database were included in the analysis. Among them, there were 593 cases in the training cohort and 255 cases in the validation cohort. The baseline characteristics of the training and validation cohorts showed no statistically significant differences (*P* > 0.05), indicating comparability (Table 1).

**Table 1:** Comparison of baseline characteristics.

Variables	Training cohort (*n* = 593)	Validation cohort (*n* = 255)	*P*-value
Age (%)			
≥35 years	445 (75.0)	193 (75.7)	0.910
<35 years	148 (25.0)	62 (24.3)	
Thyroid disorders (%)			
Yes	509 (85.8)	227 (89.0)	0.252
No	84 (14.2)	28 (11.0)	
GDM (%)			
Yes	410 (69.1)	180 (70.6)	0.735
No	183 (30.9)	75 (29.4)	
Primipara (%)			
Yes	294 (49.6)	126 (49.4)	1.000
No	299 (50.4)	129 (50.6)	
History of adverse pregnancy (%)			
Yes	300 (50.6)	124 (48.6)	0.653
No	293 (49.4)	131 (51.4)	
FHH (%)			
Yes	552 (93.1)	236 (92.5)	0.894
No	41 (6.9)	19 (7.5)	
UP (%)			
≥3+	478 (80.6)	214 (83.9)	0.502
<3+	115 (19.4)	41 (16.1)	
S/D	2.30 (2.05, 2.70)	2.32 (2.06, 2.64)	0.951
BMI (kg/m^2^)	29.0 (26.4, 31.2)	29.0 (26.2, 31.2)	0.594
MAP (mmHg)	110 (103, 117)	109 (103, 116)	0.430
WBC (10^9^/L)	9.2 (7.7, 11.5)	8.9 (7.7, 10.9)	0.138
Neut (%)	75.2 (71.0, 79.4)	74.4 (70.4, 78.6)	0.129
Lymph (%)	16.9 (14.0, 20.7)	18.2 (14.5, 21.4)	0.040
RBC (10^12^/L)	3.99 (3.70, 4.27)	4.02 (3.77, 4.32)	0.141
HGB (g/L)	120 (110, 129)	121 (112, 129)	0.403
HCT (%)	35.6 (33.0, 38.1)	36.0 (33.5, 38.2)	0.268
MCV (fl)	89.8 (85.8, 94.1)	89.7 (85.2, 93.0)	0.211
MCH (pg)	30.2 (28.6, 31.8)	30.0 (28.2, 31.4)	0.119
MCHC (g/L)	334 (326, 343)	333 (326, 342)	0.595
PLT (10^9^/L)	198 (162, 248)	204 (168, 237)	0.958
PCT (%)	0.21 (0.17, 0.25)	0.21 (0.18, 0.25)	0.795
PT (s)	11.8 (11.0, 12.5)	11.9 (11.0, 12.6)	0.580
INR	0.95 (0.91, 0.99)	0.95 (0.91, 1.00)	0.694
APTT (s)	29.8 (27.5, 32.7)	30.0 (27.7, 32.8)	0.534
TT (s)	14.9 (13.9, 15.5)	14.9 (13.9, 15.6)	0.680
FIB (g/L)	4.54 (4.08, 5.00)	4.69 (4.11, 5.17)	0.138
DD (mg/L)	1.41 (1.06, 1.96)	1.47 (1.08, 2.04)	0.576
FDP (mg/L)	4.20 (2.74, 6.49)	4.61 (2.92, 6.56)	0.341
TBIL (μmol/L)	6.6 (5.3, 8.5)	6.7 (5.3, 9.0)	0.452
TP (g/L)	56.0 (52.3, 59.3)	56.3 (53.0, 59.8)	0.173
UA (μmol/L)	370 (311, 439)	363 (307, 434)	0.775
ALP (U/L)	159 (125, 194)	165 (131, 206)	0.413
GGT (U/L)	12 (8, 18)	12 (8, 20)	0.828
LDL (mmol/L)	3.33 (2.72, 3.91)	3.25 (2.68, 3.57)	0.045
TG (mmol/L)	3.22 (2.58, 4.11)	3.10 (2.46, 3.58)	0.025
CRP (mg/L)	4.4 (2.2, 10.6)	4.6 (2.3, 10.0)	0.775
β_2_-MG (mg/L)	2.03 (1.70, 2.42)	2.06 (1.75, 2.49)	0.158
AST (U/L)	19 (15, 25)	19 (15, 26)	0.974
CR (μmol/L)	47 (41, 54)	46 (41, 55)	0.813
ALT (U/L)	11 (8, 15)	10 (8, 15)	0.557
HCY (μmol/L)	7.0 (5.8, 8.6)	7.0 (5.9, 8.1)	0.699
Ca (μmol/L)	2.07 (1.96, 2.15)	2.08 (2.00, 2.16)	0.070

Data as numbers and percentages, or median (25th–75th percentile), as appropriate.

GDM, gestational diabetes mellitus; FHH, family history of hypertension; UP, urine protein.

### Variable screening and development of the nomogram

**Figure 2:**
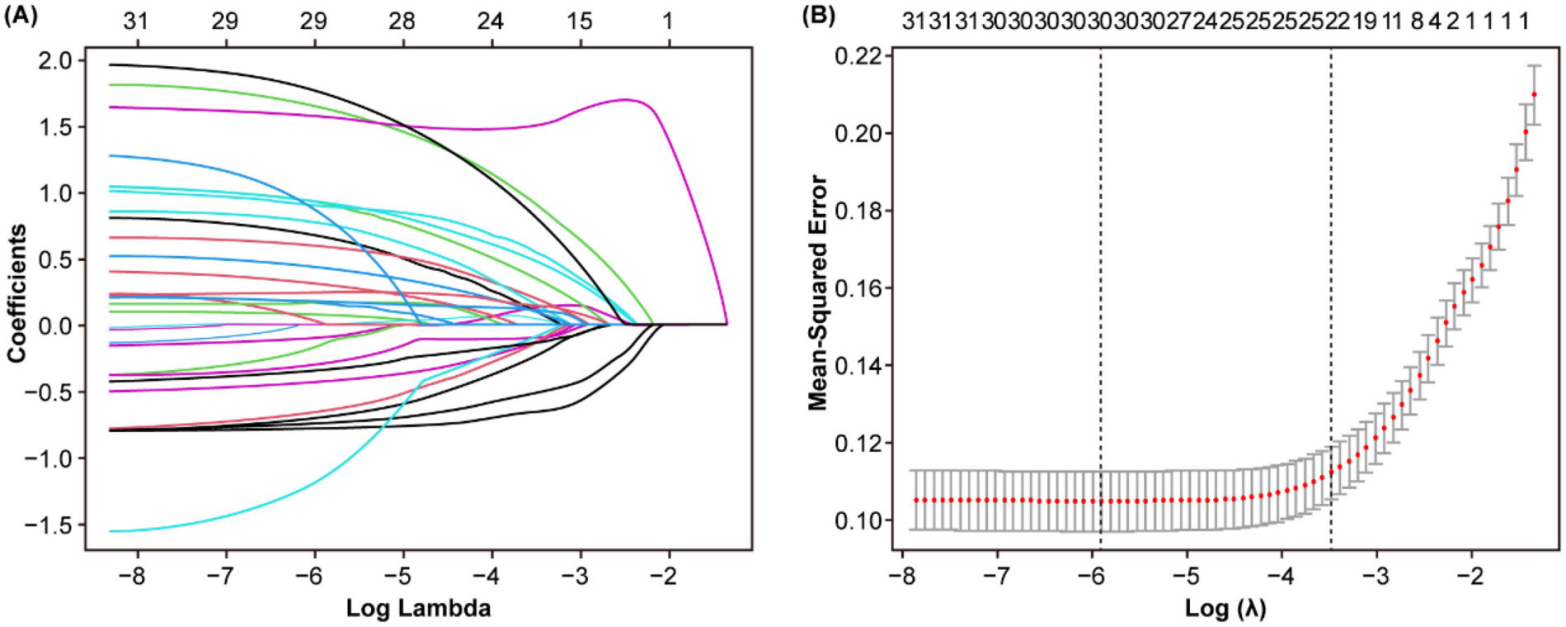
Process of feature selection in patients with SPE by LASSO logistic regression. In total, twenty-two non-zero coefficients (predictor variable) with the highest curves (AUC) for predicting SPE are selected **(A)**. The coefficients of each predictor variable are plotted vs. log (lambda) **(B)**. AUC, area under curve; LASSO, least absolute shrinkage and selection operator.

**Figure 3:**
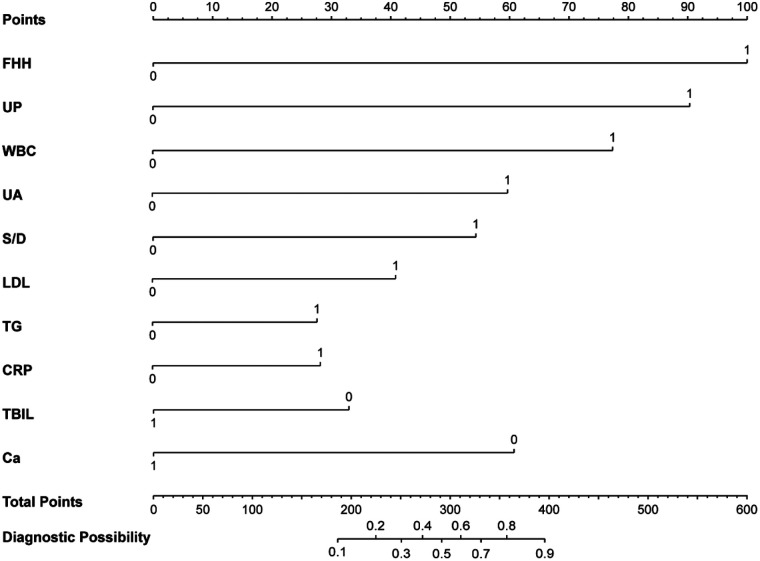
Nomogram predicting the individual probability of SPE. The points of ten variables were added to obtain the sum of points, leading to evaluate the individual probability of progression to SPE in patients with HDP. FHH, family history of hypertension; UP, urine protein.

Both non-SPE and SPE were used as dependent variables, with a total of 42 independent variables included. Due to the presence of collinearity and correlations among the independent variables, dimensionality reduction was performed to avoid model overfitting and screen for candidate predictors. Independent variables preliminarily selected through univariate analysis (*P* < 0.1) were subjected to LASSO regression analysis (Table 2). As shown in Figure 2A, the coefficients of the initially included independent variables were gradually compressed, with some eventually reduced to zero, thereby preventing model overfitting. Figure 2B demonstrates that through 10-fold cross-validation, the optimal value was selected at the minimum error within 1 standard error (*λ* = 0.031). Ultimately, 22 potential predictors for the progression of HDP to SPE were identified, including WBC, UA, TT, LDL, Ca, TBIL, TG, CRP, HGB, PCT, MAP, TP, PLT, HCY, Neut, RBC, and so on. For details, see Figure 2. To facilitate clinical application, the continuous variables selected by LASSO regression were dichotomized using the median as the cutoff point, and then incorporated into the multivariate logistic regression analysis. The results indicated that Ca, CRP, TG, LDL, UA, TBIL, WBC, S/D, UP, and FHH were significant predictors (*P* < 0.05) for the progression of HDP to SPE (Table 3). The median is as follows: WBC = 9.1 10^9^/L, UA = 368 μmol/L, S/D = 2.3, LDL = 3.28 mmol/L, TG = 3.20 mmol/L, CRP = 4.4 mg/L, TBIL = 6.7 μmol/L, Ca = 2.07 μmol/L (Appendix 1). Based on these ten predictors, a nomogram was plotted (Figure 3). According to the nomogram, the risk probability of developing severe preeclampsia can be assessed. For example, a pregnant woman with gestational hypertension or mild preeclampsia has no family history of hypertension, urine protein 1+, WBC = 8.7 10^9^/L, UA = 473 μmol/L, S/D = 2.43, LDL = 2.78 mmol/L, TG = 2.32 mmol/L, CRP = 0.1 mg/L, TBIL = 6.3 μmol/L, and Ca = 2.1 μmol/L. The risk probability of this patient progressing to severe preeclampsia is 0.168.

**Table 2:** Univariate analysis.

Variables	B	SE	OR	95% CI	*Z*	*P*-value
Ca	−1.692	0.204	0.184	0.122–0.272	−8.311	<0.001
PCT	−1.502	0.195	0.223	0.151–0.324	−7.709	<0.001
Lymph	−1.159	0.193	0.314	0.214–0.456	−6.006	<0.001
TP	−1.147	0.191	0.318	0.217–0.460	−6.007	<0.001
PLT	−1.137	0.190	0.321	0.219–0.464	−5.957	<0.001
TBIL	−1.101	0.190	0.333	0.228–0.481	−5.790	<0.001
RBC	−1.045	0.189	0.352	0.241–0.507	−5.520	<0.001
HGB	−0.815	0.186	0.443	0.306–0.635	−4.389	<0.001
HCT	−0.703	0.184	0.495	0.344–0.709	−3.815	<0.001
INR	−0.541	0.181	0.582	0.407–0.829	−2.991	0.003
GDM	−0.306	0.200	0.736	0.495–1.083	−1.535	0.125
MCHC	−0.243	0.180	0.784	0.551–1.115	−1.353	0.176
ALP	−0.204	0.180	0.815	0.572–1.159	−1.137	0.255
APH	−0.191	0.180	0.826	0.580–1.174	−1.066	0.287
PT	−0.140	0.179	0.870	0.611–1.236	−0.779	0.436
BMI	−0.124	0.179	0.884	0.621–1.256	−0.690	0.490
FIB	−0.040	0.179	0.961	0.675–1.365	−0.224	0.823
Primipara	0.104	0.179	1.11	0.781–1.579	0.582	0.560
Thyroid disorders	0.180	0.251	1.197	0.724–1.942	0.715	0.475
MCH	0.205	0.180	1.228	0.863–1.750	1.138	0.255
Age	0.234	0.203	1.264	0.845–1.875	1.153	0.249
GGT	0.312	0.181	1.367	0.960–1.952	1.728	0.084
DD	0.444	0.181	1.559	1.095–2.226	2.457	0.014
MCV	0.451	0.181	1.570	1.103–2.244	2.492	0.013
FDP	0.638	0.182	1.892	1.326–2.711	3.500	<0.001
CRP	0.747	0.184	2.110	1.475–3.036	4.059	<0.001
ALT	0.759	0.190	2.136	1.477–3.119	3.987	<0.001
HCY	0.801	0.186	2.228	1.552–3.222	4.304	<0.001
APTT	0.810	0.185	2.247	1.568–3.244	4.372	<0.001
TT	0.957	0.189	2.605	1.806–3.794	5.062	<0.001
TG	0.970	0.189	2.639	1.831–3.838	5.146	<0.001
AST	1.045	0.189	2.844	1.971–4.144	5.520	<0.001
LDL	1.125	0.192	3.079	2.126–4.508	5.872	<0.001
Neut	1.215	0.195	3.37	2.314–4.971	6.237	<0.001
MAP	1.215	0.195	3.37	2.314–4.971	6.237	<0.001
CR	1.258	0.193	3.518	2.421–5.173	6.504	<0.001
UA	1.381	0.197	3.977	2.719–5.896	7.004	<0.001
S/D	1.407	0.205	4.086	2.757–6.163	6.871	<0.001
WBC	1.495	0.205	4.46	3.009–6.732	7.292	<0.001
β_2_-MG	1.520	0.199	4.573	3.118–6.804	7.651	<0.001
FHH	1.753	0.349	5.772	2.969–11.790	5.023	<0.001
UP	2.985	0.266	19.78	11.950–34.000	11.230	<0.001

GDM, gestational diabetes mellitus; APH, history of adverse pregnancy; FHH, family history of hypertension; UP, urine protein.

**Table 3:** Multivariate logistic regression analysis.

Variables	B	SE	OR	95% CI	*Z*	*P*-value
Ca	−0.866	0.325	0.420	0.220–0.791	−2.668	0.008
PCT	−0.808	0.445	0.446	0.182–1.049	−1.815	0.070
TBIL	−0.739	0.299	0.477	0.263–0.855	−2.472	0.013
PLT	−0.638	0.460	0.528	0.216–1.321	−1.387	0.165
HGB	−0.602	0.361	0.548	0.269–1.112	−1.667	0.096
TP	−0.406	0.310	0.666	0.362–1.226	−1.308	0.191
Lymph	−0.265	0.490	0.767	0.292–2.001	−0.542	0.588
RBC	−0.099	0.365	0.905	0.441–1.853	−0.272	0.785
β_2_-MG	0.028	0.322	1.028	0.543–1.924	0.086	0.932
AST	0.172	0.311	1.188	0.643–2.182	0.554	0.579
Neut	0.258	0.495	1.294	0.489–3.423	0.521	0.602
MAP	0.502	0.300	1.653	0.918–2.989	1.676	0.094
TT	0.541	0.301	1.718	0.953–3.118	1.795	0.073
CRP	0.649	0.311	1.914	1.044–3.552	2.086	0.037
TG	0.717	0.304	2.048	1.133–3.753	2.354	0.019
LDL	0.900	0.304	2.460	1.364–4.505	2.965	0.003
S/D	1.063	0.304	2.895	1.609–5.326	3.495	<0.001
UA	1.119	0.327	3.060	1.623–5.879	3.419	0.001
UP	1.555	0.391	4.734	2.224–10.350	3.977	<0.001
WBC	1.782	0.344	5.945	3.084–11.910	5.187	<0.001
FHH	1.802	0.549	6.062	2.092–18.130	3.280	0.001

FHH, family history of hypertension; UP, urine protein.

### Validation of the nomogram

**Figure 4:**
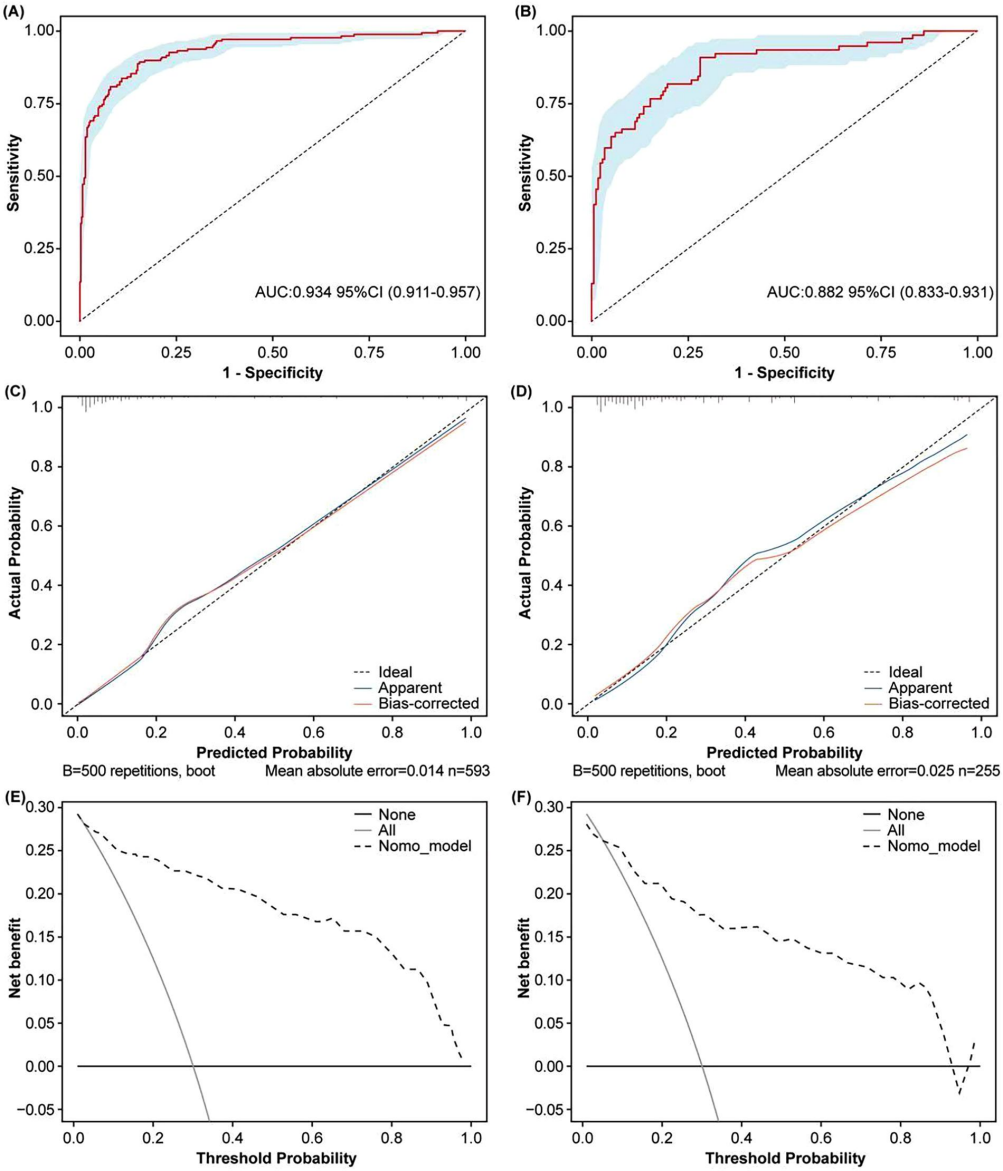
Validation of the nomogram. Receiver operating characteristic (ROC) curve of the nomogram in the training cohort **(A)**. ROC curve of the nomogram in the validation cohort **(B)**. Calibration curve of the nomogram for the training cohort **(C)**. Calibration curve of the nomogram for the validation cohort **(D)**. Decision curve analysis (DCA) of the nomogram in the training cohort **(E)**. DCA of the nomogram in the validation cohort **(F)**.

The results of ROC curve analysis showed that the AUCs were 0.934 in the training cohort and 0.882 in the validation cohort (Figures 4A,B), indicating that the model exhibited good discrimination ability.

Calibration charts were plotted to evaluate the consistency between the predicted and actual probability of SPE in both the training and validation cohorts (Figures 4C,D). The calibration curves closely aligned with the ideal curves in both cohorts, indicating that our model had good consistency between the prediction result and actual observation.

Moreover, the DCA plots demonstrated that when the probability threshold ranges from 5% to 95% and 10% to 95%, the predictive model demonstrates a clinical net benefit rate for SPE events that is higher than both the “intervention-for-all” and “no-intervention” strategies in both the training and validation cohorts (Figures 4E,F).

### Development and validation of machine learning models

**Figure 5:**
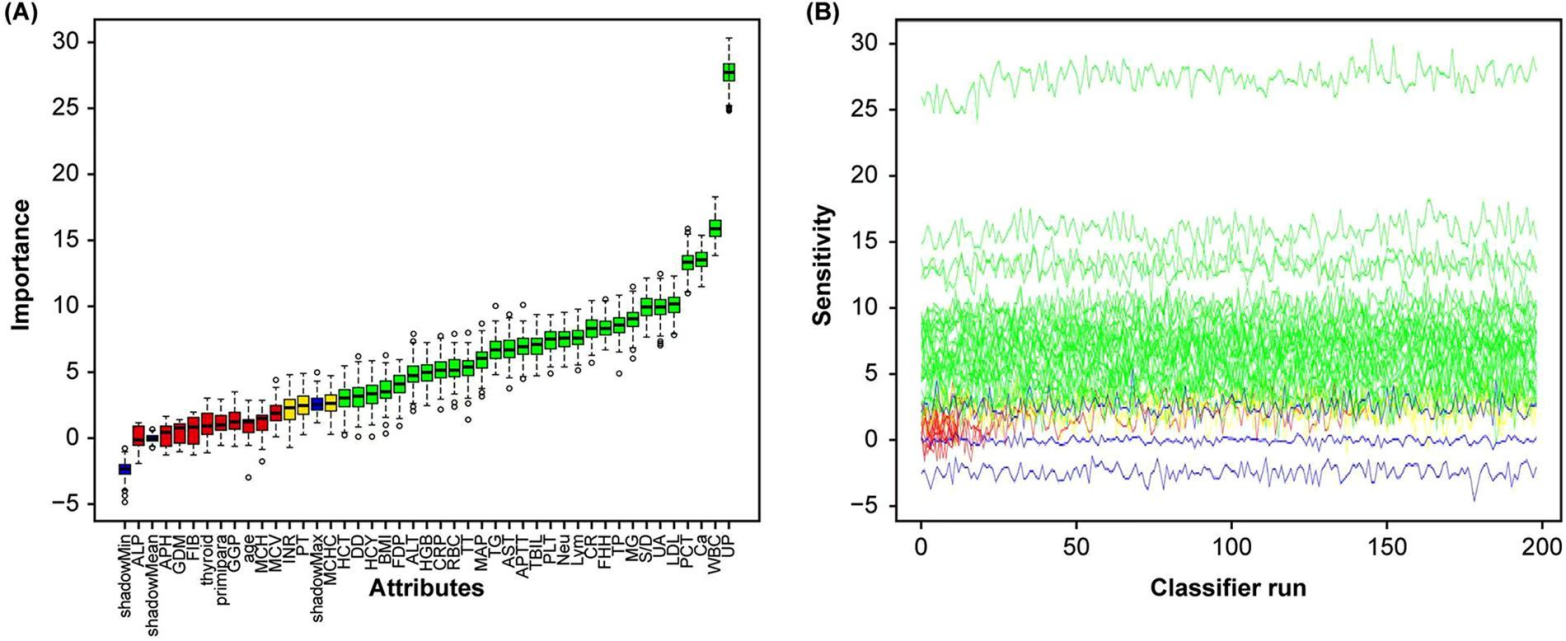
Feature selection for the potential markers associated with endpoints using the boruta algorithm. The process of feature selection **(A)**. The value evolution of *Z*-score in the screening process **(B)**. The horizontal axis shows the name of variables and the number of iterations in **(A,B)**, respectively. While the vertical axis represents the *Z*-value of each variable, and the blue boxes and lines corresponds to the minimum, average, and maximum *Z*-scores for a shadow feature. The green boxes and lines represent confirmed variables, the yellow ones represent tentative attributes, and the red ones represent rejected variables in the model calculation.

**Figure 6:**
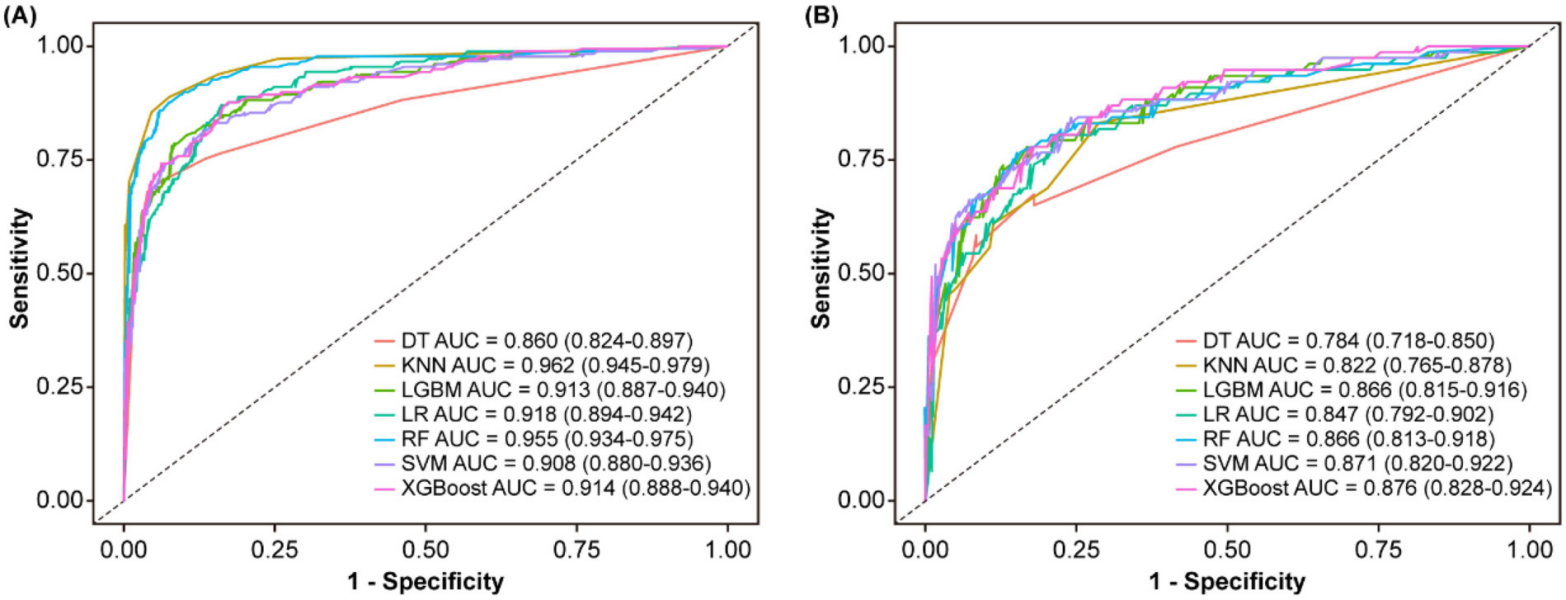
Comparison of ROC across different models**.** The ROC curves comparison of the seven models in training cohort **(A)**. The ROC curves comparison of the seven models in validation cohort **(B)**. DT, decision trees; KNN, K-nearest neighbors; LGBM, LightGBM; SVM, support vector machines; RF, random forest; LR, logistic regression.

The results showed that after 200 iterations, continuously removing features it deemed unimportant, the maximum shadow feature clearly distinguished between important and non-important features. A total of 29 variables (UP, WBC, Ca, PCT, S/D, UA, TP, CR, FFH, Lymph, Nuet, PLT, etc.) were identified as important features. Three variables (MCHC, PT, INR) were identified as suspiciously important variables, while 11 variables were rejected (Figure 5A). The line chart primarily illustrates parameter changes (Figure 5B), with each iteration yielding an importance score. By comparing the importance scores of different variables, we can evaluate which variables are more critical for predicting SPE. Ultimately, the results should be interpreted based on the box plot (Figure 5A). For comparison with the nomogram model, this study selected the top ten most important variables identified through Boruta screening to construct six machine learning models: DT, LightGBM, SVM, XGBoost, RF, and KNN. Among these, the XGBoost model demonstrated superior performance, achieving the following metrics in the validation cohort: an area under the receiver operating characteristic curve (AUC) of 0.876 (95% CI: 0.828–0.924), accuracy of 80.8%, sensitivity of 78.0%, specificity of 82.0%, positive predictive value of 65.2%, and negative predictive value of 89.6%. Compared to the logistic regression (LR) model, the XGBoost model exhibited a higher AUC, accuracy, and sensitivity (Figure 6, Table 4), thereby reducing the clinical misdiagnosis rate.

**Table 4:** Comparison of performances of seven models in the validation cohort.

Model	AUC	ACC%	SEN%	SPE%	PPV%	NPV%	*P*-value
XGBOOST	0.876 (0.828–0.924)	0.808	0.780	0.820	0.652	0.896	<0.001
KNN	0.822 (0.765–0.878)	0.804	0.610	0.888	0.702	0.840	<0.001
RF	0.866 (0.813–0.918)	0.820	0.753	0.848	0.682	0.888	<0.001
LGBM	0.866 (0.815–0.916)	0.828	0.623	0.916	0.762	0.849	<0.001
SVM	0.871 (0.820–0.922)	0.824	0.727	0.865	0.646	0.861	<0.001
DT	0.784 (0.718–0.850)	0.808	0.701	0.865	0.700	0.880	<0.001
LR	0.847 (0.792–0.902)	0.792	0.688	0.890	0.747	0.870	<0.001

95% confidence intervals were included in brackets.

ACC, accuracy; AUC, area under the curve; DT, decision trees; KNN, K-nearest neighbors; LGBM, LightGBM; SVM, support vector machines; RF, random forest; LR, logistic regression; NPV, negative predictive value; PPV, positive predictive; SEN, sensitivity; SPE, specificity.

### Model interpretation

**Figure 7:**
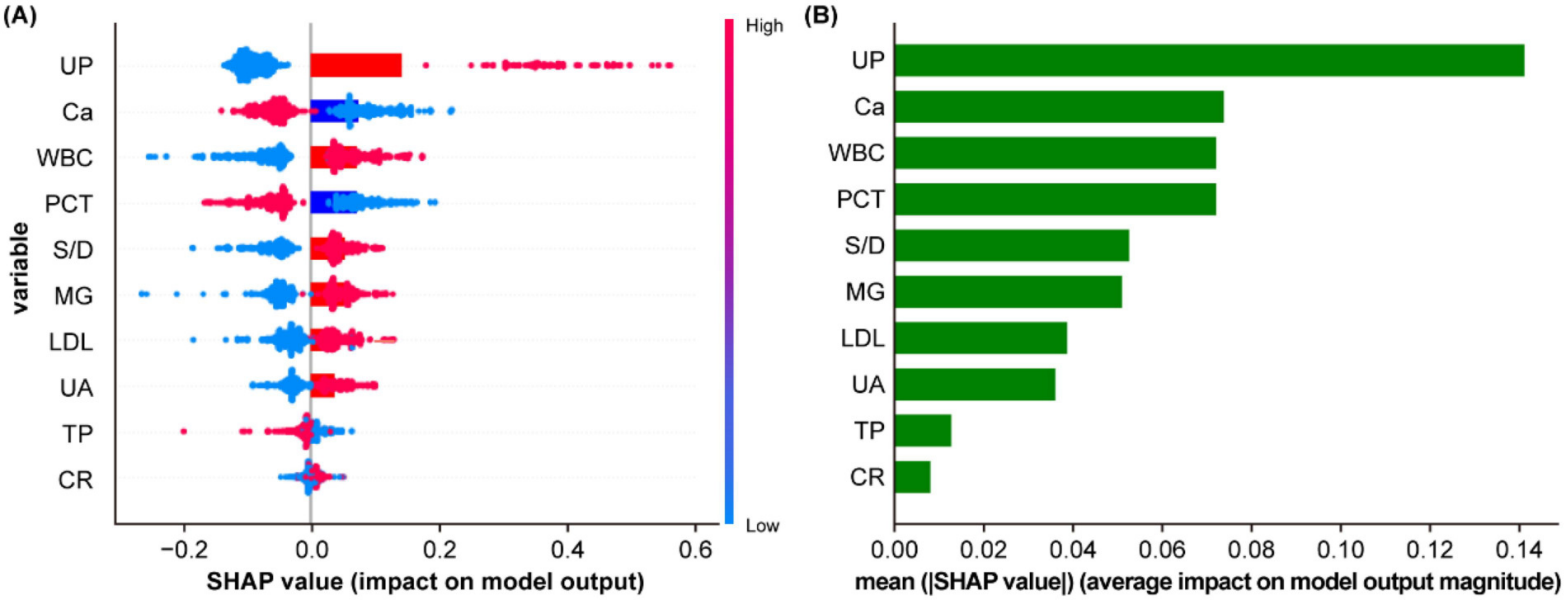
XGBoost model based on the SHAP algorithm. Feature attributes in the black-box model. Each line represents a feature, and the *x*-axis represents SHAP values, indicating the impact of the feature on the outcome. Each point represents a sample. The redder the colour, the larger the feature value; the blue the colour, the smaller the feature value **(A)**; Feature importance ranking indicated by SHAP **(B)**.

To ensure a comprehensive understanding of the selected variables, we employed the SHAP algorithm to highlight their predictive importance in the optimal XGBoost model for SPE. Figure 7A visually demonstrates the 10 key features of the XGBoost model, including UP, Ca, WBC, PCT, S/D, MG, LDL, UA, TP and CR. The influence of each feature is illustrated by uniquely colored dots: red indicating higher risk values and blue indicating lower ones. Figure 7B depicts the importance of each factor. According to the ranking by SHAP values, UP contributes the most to the model, followed by Ca, WBC, PCT, S/D, MG, LDL, UA, CR.

## Discussion

We developed a nomogram model designed to predict the risk of SPE among pregnant women diagnosed with hypertensive disorders. The model's key performance characteristics discrimination, calibration, and clinical utility were assessed using ROC curves, calibration curves, and DCA curves, respectively. Furthermore, we constructed machine learning models. This study expands on the exploration of machine learning-based predictive models, including the XGBoost model, KNN model, RF model, LightGBM model, SVM model, and DT model. By comparing the performance of models built with these diverse machine learning algorithms and evaluating their efficacy across both the training and validation cohorts, the XGBoost model clearly demonstrated superior performance. The performance comparison in the validation cohort showed that the XGBoost model exhibited higher accuracy, area under the receiver operating characteristic curve (AUC), sensitivity, and negative predictive value [80.8%, 0.876 (95% CI: 0.828–0.924), 78.0%, 89.6%] compared to the logistic regression model [79.2%, 0.847 (95% CI: 0.792–0.902), 68.8%, 87.0%].

Currently, machine learning has been widely applied in the medical field, with researchers both domestically and internationally utilizing machine learning algorithms to establish predictive models for preeclampsia (PE) (15, 16). However, predictive models specifically targeting the progression of this disease to its severe forms remain scarce. Deng Xingyu et al. (17) found that using PE risk factors alone resulted in low model performance. By combining risk factors with laboratory indicators to construct LR and XGBoost models, the XGBoost model showed significant improvements in AUC, specificity, and sensitivity. Zheng Jiangyuan et al. (18) conducted a retrospective study of 1,609 pregnant women, constructing LR and LightGBM models using 13 influencing factors of PE; the LightGBM model demonstrated superior predictive performance over the LR model. Villalaín et al. (19) compared KNN and SVM algorithms in constructing predictive models for early-onset preeclampsia, concluding that the SVM algorithm offered better predictive efficacy. Due to the diversity of predictive factors and regional differences in study populations, disease prediction models exhibit considerable complexity and variation.

Our study found that when comparing machine learning models with the multifactorial logistic regression model, both demonstrated similar predictive performance in the training cohort. However, in the validation cohort, the XGBoost model exhibited a higher area under the receiver operating characteristic curve, accuracy, and sensitivity, while the multifactorial logistic regression model showed higher specificity. Nevertheless, the classic multifactorial logistic model remains well-established and interpretable, and utilizes a nomogram as a visual tool for prediction. This provides clinicians with an intuitive and convenient method. By calculating a total score based on patients' baseline data, the probability of each HDP patient progressing to SPE can be predicted. Conversely, the prediction model based on the XGBoost algorithm demonstrated high discriminative ability, enabling the early prediction of HDP disease progression, which is of significant importance for timely treatment.

By comparing the factors utilized in both the machine learning models and the nomogram model, our study identified six common predictive factors, including urine protein, WBC, UA, Ca, umbilical artery S/D ratio, and LDL. This finding validates their collective roles as warning indicators for the progression of HDP SPE, suggesting that combining both approaches may offer greater clinical value than using either model alone. In both the nomogram and machine learning models, urine protein was consistently identified as the factor contributing most significantly to prediction. It is reported that the incidence of proteinuria during pregnancy is 8% (20, 21). In SPE, proteinuria results from glomerular enlargement, endothelial cell swelling, and fibrin deposition, leading to the leakage of plasma proteins from the glomeruli. Although proteinuria has been considered a sufficient but necessary condition for diagnosing PE since 2013, its prognostic value remains high. A study by Tzur et al. (22) noted that among 165 pregnant women diagnosed with proteinuria, 38 (23%) developed SPE, and further found that every 500 mg/day increase in proteinuria raised the risk of progressing to SPE by 14%. These findings strongly support that proteinuria is a critical high-risk factor for the progression of HDP to SPE. Consequently, for pregnant women presenting with proteinuria ≥3+, 24-h urine protein testing should be conducted to accurately assess the risk of HDP progression, enabling necessary clinical intervention.

However, this study still has some limitations. It was a single-center retrospective study with a moderate sample size. Further research should expand the clinical sample size and conduct multicenter prospective studies to enhance the clinical feasibility of the predictive model.

## Data Availability

The original contributions presented in the study are included in the article/Supplementary Material, further inquiries can be directed to the corresponding authors.

## Appendix 1

**Table A1:** Binary classification assignment translation.

Variables	Assignment
Ca	≥2.07 = 1, <2.07 = 0
AST	≥19 = 1, <19 = 0
β2-MG	≥2.035 = 1, <2.035 = 0
CRP	≥4.4 = 1, <4.4 = 0
TG	≥3.2 = 1, <3.2 = 1
LDL	≥3.28 = 1, <3.28 = 0
UA	≥367.85 = 1, <367.85 = 0
TP	≥56.1, <56.1 = 0
TBIL	≥6.65 = 1, <6.65 = 0
TT	≥14.9 = 1, <14.9 = 0
PCT	≥0.21 = 1, <0.21 = 0
PLT	≥199 = 1, <199 = 0
HGB	≥120.5 = 1, <120.5 = 0
RBC	≥4.01 = 1, <4.01 = 0
Lymph	≥17.3 = 1, <17.3 = 0
Neut	≥74.9 = 1, <74.9 = 0
WBC	≥9.1 = 1, <9.1 = 0
MAP	≥109.67 = 1, <109.67 = 0
Umbilical artery blood flow S/D	≥2.3 = 1, <2.3 = 0
